# Influence of Cellular Composition and Exogenous Activation on Growth Factor and Cytokine Concentrations in Canine Platelet-Rich Plasmas

**DOI:** 10.3389/fvets.2017.00040

**Published:** 2017-04-05

**Authors:** Samuel P. Franklin, Kate E. Birdwhistell, Alena Strelchik, Bridget C. Garner, Benjamin M. Brainard

**Affiliations:** ^1^Department of Small Animal Medicine and Surgery, College of Veterinary Medicine, University of Georgia, Athens, GA, USA; ^2^Regenerative Bioscience Center, University of Georgia, Athens, GA, USA; ^3^Department of Pathology, College of Veterinary Medicine, University of Georgia, Athens, GA, USA

**Keywords:** canine, platelet-rich plasma, platelet activation, growth factors, cytokines

## Abstract

**Objective:**

The purposes of this study were to (1) evaluate correlations among platelet, leukocyte, growth factor, and cytokine concentrations in canine platelet-rich plasmas (PRPs) produced from five different canine PRP-concentrating systems and (2) compare the effects of different activation protocols on platelet activation and growth factor release from one of these PRPs.

**Methods:**

PRP was made using blood from 15 dogs and each of 5 different PRP systems in a cross-over design. Complete blood counts were performed to quantify platelet and leukocyte concentrations. PRPs were activated, or not, according to manufacturer instructions, and transforming growth factor-β1 (TGF-β1), platelet-derived growth factor-BB (PDGF-BB), vascular endothelial growth factor, and tumor necrosis factor-alpha (TNF-α) were quantified. Differences among platelet, leukocyte, and growth factor concentration were compared among the different systems. Correlations between platelet and anabolic growth factor concentrations were assessed. Subsequently, PRP was made from 12 additional dogs using one of the devices. Each PRP was divided into three aliquots that were activated with calcium chloride (CaCl_2_), human γ-thrombin (HGT), or not activated. Expression of CD62P and platelet-bound fibrinogen (CAP1) was quantified for each activation group. Concentrations of TGF-β1, PDGF-BB, and TNF-α were also quantified for each activation group and a fourth group that was frozen/thawed. Differences among activation groups were assessed by a Friedman test.

**Results:**

There were statistically significant differences among the PRPs made with difference devices with regard to platelet, leukocyte, TGF-β1, and PDGF-BB concentrations (*p* < 0.0001). There were weak to moderate correlations (*R*^2^ = 0.07–0.58) between platelet and anabolic growth factor concentrations but it appeared that activation had a greater effect on growth factor concentration than did cellular composition. Intentional platelet activation significantly increased CD62P and CAP1 expression as well as TGF-β1 and PDGF-BB concentrations in the one PRP in which all activation methods were assessed. Activation with HGT resulted in the greatest platelet activation, and CaCl_2_ and freeze/thaw elicited moderate increases in either growth factor release or CD62P and CAP1 expression.

**Conclusion:**

There are positive correlations between platelet and anabolic growth factor concentrations in canine PRPs. However, intentional platelet activation has a greater effect on growth factor delivery than platelet concentration. Thrombin provides more robust activation than CaCl_2_.

## Introduction

Human and equine platelet-rich plasmas (PRPs) that are prepared with different methodologies vary notably in their cellular composition, growth factor concentrations, and catabolic cytokine concentrations (1–4). Furthermore, it has been shown that the concentration of transforming growth factor-β1 (TGF-β1) and PDGF-AB is significantly and positively correlated with the platelet concentration in human and equine PRPs (4, 5). Conversely, the catabolic cytokines interleukin-1β (IL-1β) and TNF-α were positively correlated with the leukocyte concentration in two different human PRP preparations (4). Given that one of the primary mechanisms by which PRP may be beneficial is the delivery of anabolic growth factors, balanced by the delivery of catabolic cytokines, variability in the concentration of these proteins provided by different PRPs may be clinically relevant (6). Indeed, there is a growing body of evidence that variations in cellular composition, growth factor concentrations, and inflammatory cytokine concentrations in PRP can influence tissue metabolism both *in vitro* and *in vivo* (7–11).

As with human and equine PRPs, studies have demonstrated that canine PRPs also vary dramatically in their cellular composition (12–16). In addition, three studies provide data on growth factor concentration(s) in three different canine PRPs (13, 17, 18). However, just one PRP preparation was assessed in each study, cytokine concentrations were not quantified, and assessment of possible associations between cellular composition and growth factor concentrations in different canine PRPs has not been performed. As a result, there remains a need to quantify growth factor and cytokine concentrations in different canine PRPs in order to fully characterize these products and to assess the relative impact of cellular composition on growth factor and cytokine delivery.

In addition to cellular composition of PRPs, exogenous platelet activation protocols have been shown to affect growth factor and cytokine concentrations in PRPs and may, therefore, influence clinical efficacy (4, 17, 19–21). Studies comparing the effects of different platelet activation protocols on PRPs provide inconsistent results (22, 23). One study demonstrated greater cumulative growth factor elution from human PRP with collagen activation than with bovine thrombin activation (23). Conversely, another study reported greater growth factor release from human PRP with use of bovine thrombin when compared to collagen activation (22). In horses, exposure of PRP to *ex vivo* collagen or fibrin resulted in relatively small increases in the production of platelet-derived growth factor-BB (PDGF-BB) and TGF-β1 (21, 24). Exposure of equine PRP to calcium chloride (CaCl_2_) and bovine thrombin produced more dramatic increases in PDGF-BB and TGF-β1 (21, 24).

With regard to canine PRPs, evaluation of calcium gluconate activation, both with and without batroxobin, resulted in increased TGF-β1 concentrations in comparison to PRP that was not activated (19). More recently, CD62P expression was shown to be significantly increased in a leukocyte-rich canine PRP after activation with bovine thrombin (25). The CD62P protein, or P-selectin, is bound to the platelet alpha granule that remains internalized in platelets until platelets are activated and the alpha granule fuses with the platelet external membrane at which time the CD62P protein is externalized (26). Hence, increased CD62P expression is associated with activation and degranulation of platelets and is used as a marker of platelet activation (26, 27).

Although the aforementioned studies in dogs provide valuable information, there remain unanswered questions regarding activation of canine PRPs. Notably, it remains unclear what effect untested activation protocols, such as use of CaCl_2_, have on canine platelet activation. Further, only concentrations of TGF-β1 have been assessed and the effects of activation on concentrations of other growth factors and inflammatory cytokines have not been described. Last, comparison of calcium-based activation versus the use of thrombin has not been performed using canine PRP (21, 28, 29).

The purposes of this study were to (1) characterize the specific leukocyte populations in PRPs prepared using five different commercially available systems; (2) characterize the anabolic growth factor and tumor necrosis factor-α (TNF-α) concentrations in those PRPs; (3) assess whether there was a correlation between the platelet or leukocyte concentrations of the PRPs and the growth factor and TNF-α concentrations; and (4) quantify the effects of using CaCl_2_ or human γ-thrombin (HGT) on platelet activation and growth factor concentrations in one of these canine PRPs. It was hypothesized that leukocyte concentrations would differ between PRPs. In addition, like previous studies using human and equine PRPs, it was hypothesized that there would be a moderate positive correlation (*R*^2^ > 0.5) between PRP platelet concentrations and PRP anabolic growth factor concentrations, as well as a moderate (*R*^2^ > 0.5) positive correlation between PRP leukocyte concentrations and TNF-α concentrations (4, 5). It was hypothesized that all activation protocols would increase expression of CD62P and platelet-bound fibrinogen as well as concentrations of the anabolic growth factors TGF-β1 and PDGF-BB.

## Materials and Methods

This study was reviewed and approved by the University of Georgia Clinical Research Committee. Written and verbal description of the study was provided to owners prior to enrollment and all owners signed written consent forms upon enrollment. No additional vulnerable populations were involved in this study.

### Animals

Fifteen healthy dogs weighing at least 15 kg and free of medical problems, other than osteoarthritis, as determined on the basis of medical history and results of a general physical examination and a complete blood count (CBC) were included. Results of the CBC had to be normal, including platelet counts greater than 125 × 10^3^ platelets/μL and less than 600 × 10^3^ platelets/μL. No dogs received medication for the duration of the study period except for one dog that received the same daily dose of Carprofen throughout the study period.

### PRP Systems

Platelet-rich plasma was made using each of five different commercially available systems in a 15-dog cross-over study (12). Five commercially available systems were used for PRP preparation: System 1 (Protec PRP, PulseVet, Alpharetta, GA, USA), System 2 (MediVet PRP, MediVet Biologics, Nicholasville, KY, USA), System 3 (C-PET, Pall Corp, Port Washington, NY, USA), System 4 (SmartPReP 2, Harvest Technologies, Plymouth, MA, USA), and System 5 (Angel, Arthrex Vet Systems, Naples, FL, USA). Four of these systems (Systems 1, 2, 4, and 5) involved use of centrifugation, and one (System 3) was a filtration-based system.

### Blood Acquisition

Blood was acquired from each dog on three occasions for PRP preparation with a minimum 4-week interval between successive phlebotomies. Dogs were sedated for all blood draws by IV administration of dexmedetomidine (Dexdomitor, Zoetis, Florham Park, NJ, USA) (0.005 mg/kg) and nalbuphine hydrochloride (Hospira, Lake Forest, IL, USA) (0.5 mg/kg). Sedated dogs were placed in lateral recumbency, and blood was collected from a jugular vein. PRP was prepared from each dog for all five systems. PRPs were made using Systems 1 and 2 associated with the first phlebotomy, with System 3 being used 4 weeks later, and with Systems 4 and 5, another 4 weeks later.

Blood was collected using an evacuated container needle and pressurized blood tubes containing anticoagulant (sodium citrate or ACD-A) that were provided by the manufacturer of Systems 1 and 2. There was one blood tube in the kit for System 1, and it was filled in accordance with the manufacturer instructions. All four blood tubes included with System 2 were filled (total volume, 40 mL) even though manufacturer instructions stated that only two blood tubes (total volume, 20 mL) were needed when preparing PRP from canine blood. When collecting blood for use with Systems 3, 4, and 5, an 18-gauge, 45-mm catheter was inserted into a jugular vein, and blood was collected into a 60-mL syringe containing ACD-A. Blood volumes of 55, 29, and 44 mL were added to 5, 3, and 6 mL of ACD-A for systems 3, 4, and 5, respectively.

Blood tubes were manually inverted several times immediately following blood acquisition, and then placed on a blood tube rocker for 5 min to thoroughly mix the blood and anticoagulant. A 2-mL aliquot of anticoagulated whole blood was then moved to a tube coated with dry EDTA; that tube was maintained on a blood tube rocker to thoroughly mix the blood and EDTA. Tubes remained on the blood tube rocker until analysis.

### PRP Preparation and Sample Processing

Platelet-rich plasma was then made using the remaining anticoagulated whole blood and manufacturer-provided materials and centrifuges in accordance with manufacturer instructions, except with system 2 in which the manufacturer-provided centrifuge was not used. Rather, a swinging bucket centrifuge (Sorvall Legend X1R centrifuge with a TX-400 rotor, Thermo Fisher Scientific, Waltham, MA, USA) (radius 16.8 cm) was used with the acceleration and deceleration both set at 4 (scale of 1 to 10). For System 5, the desired hematocrit in the PRP was selected as 2%. After the PRP was made, an aliquot (1–2 mL) of PRP was collected and placed in a blood tube coated with dry EDTA, mixed on a blood tube rocker, and a CBC was performed. Smears of both the whole blood and the PRP were made for differential analyses.

After aliquots of the PRP were taken for performance of the CBC and differentials, the remaining volume of the PRP samples were either activated (or not) according to manufacturer instructions. Specifically, both Systems 1 and 2 include a CaCl_2_ activator. The authors were informed that this activator did not need to be used for System 1 (Adrian Lock, PulseVet, Alpharetta, GA, USA personal communication, May 2014). Therefore, no activation was performed for PRPs from Systems 1, 3, 4, and 5. These PRPs were centrifuged at 20,000 × *g* for 10 min to pellet any remaining cells in the PRP. The supernatant was isolated and frozen at −80°C for future growth factor and cytokine analysis as per manufacturer recommendations and as has been done with previous quantification of growth factor concentrations in equine PRP (21). The PRP from System 2 was activated by mixing the PRP (2 mL) with CaCl_2_ (Acticell, MediVet Biologics, Nicholasville, KY) to a final CaCl_2_ concentration of 24 mg/mL. After 30–45 min of mixing with the activator, the remaining liquid portion (releasate) was collected and frozen at −80°C for future growth factor and cytokine analysis.

### Whole Blood and PRP Analysis

All samples of whole blood and PRP were maintained on a blood tube rocker until performance of a CBC using an automated hematology analyzer (HemaTrue, Heska Corp, Loveland, CO, USA) that quantified the hematocrit, leukocyte, RBC, and platelet concentrations. All samples were analyzed 15–60 min after sample collection. A blood smear of each sample was prepared for a manual differential count and assessment of platelet clumping. One hundred leukocytes were counted for all samples except for some of the leukocyte-poor PRPs in which the differential count was based upon assessment of 25 cells.

### Growth Factor and Cytokine Quantification

The PRP supernatants or releasates (System 2) were thawed at room temperature and TGF-β1 (Mouse/Rat/Porcine/Canine TGF-β1 Quantikine ELISA Kit, R&D Systems, Minneapolis, MN, USA), PDGF-BB (Human PDGF-BB Immunoassay, R&D Systems, Minneapolis, MN, USA), vascular endothelial growth factor (VEGF; Canine VEGF Immunoassay, R&D Systems, Minneapolis, MN, USA), and TNF-α (Canine TNF-α Immunoassay, R&D System, Minneapolis, MN, USA) were quantified using enzyme-linked immunosorbent assays (ELISAs). With the exception of the PDGF-BB ELISA, these assays have been validated by the manufacturers for use with canine plasma samples. Further, all of the ELISAs, including the PDGF-BB assay, have been shown to meet industry accepted standards of both intra and inter-assay precision with ACD-A anticoagulated canine plasma samples specifically (30). Samples were run in duplicate, and all samples were assessed on the same day (simultaneously) for a given growth factor or TNF-α. Manufacturer instructions were followed including all instructions regarding sample acid-activation for the TGF-β1 ELISA. Following manufacturer instructions, samples were diluted 60-, 20-, and 2-fold for TGF-β1, PDGF-BB, and VEGF assays, respectively. Samples for the TNF-α assay were run without dilution as instructed by the manufacturer. The PDGF-BB ELISA is a human ELISA kit that contains a human recombinant standard. For this study, a canine recombinant PDGF-BB standard (PDGF-BB Canine Recombinant Protein, Genorise, Glen Mills, PA, USA) was used (30). All sample concentrations were assessed using a four-parameter logistic equation generated using statistical software (GraphPad, Prism 6, La Jolla, CA, USA).

### Data Analysis

Distribution of the platelet, leukocyte, growth factor, and cytokine concentrations were assessed for normality using histograms, probability plots, and multiple tests for normality including a Shapiro–Wilk test (SAS version 9.4, Cary, NC, USA). The platelet and total leukocyte data were adequately normal to justify comparison among PRP systems using a linear mixed effects model (SAS version 9.4, Cary, NC, USA). The full model included a fixed factor for the system and a random intercept for each dog. Pair-wise comparisons were made using a Tukey’s test.

Data for TGF-β1 and PDGF-BB concentrations were not normally distributed so differences among PRP preparations using different systems were compared using a non-parametric Friedman test (SAS version 9.4, Cary, NC, USA). When significant, multiple comparisons were performed using Conover’s test [R (R Core Team); http://www.R-project.org/]. A Bonferroni correction was used to adjust for multiple comparisons.

Correlation between platelet concentration and TGF-β1 concentration was assessed for those PRPs that were not activated (Systems 1, 3, 4, 5). Correlations between platelet concentration and both TGF-β1 and PDGF-BB concentrations were assessed for the PRP releasates from System 2 that had been activated. All correlations were assessed using a Pearson’s test.

### Assessment of Platelet Activation

#### Animals

In order to compare the effects of different activation protocols on canine PRP, blood was collected from an additional 12 healthy dogs that were not receiving any medications other than monthly paraciticides. Dogs were required to weigh at least 15 kg.

#### Blood Acquisition

Fresh whole blood (52.8 mL) was acquired from each dog without sedation. Blood was obtained from a jugular vein using a 19-gauge butterfly catheter, and was collected into syringes pre-loaded with ACD-A such that all blood was anticoagulated in a 1:7 ratio of ACD-A to whole blood. Syringes were placed on a platform rocker on a gentle setting for 5 min to thoroughly mix the blood and ACD-A prior to PRP preparation. For each dog, 1 mL of anticoagulated whole blood was analyzed using an automated hematology analyzer (HemaTrue, Heska Corp, Loveland, CO, USA) for a CBC. For nine dogs, the prothrombin time, activated partial thromboplastin time, and fibrinogen values were also quantified, it was done on citrated blood, collected separately.

#### Plasma Preparation

Fifty milliliters of the anticoagulated whole blood were processed using a commercially available device (Angel, Arthrex Vet Systems, Naples, FL, USA; System 5) to produce PRP. PRP was created using the “manual” setting in which the user visually differentiates and manually distributes the resultant product into separate PRP, platelet-poor plasma, and red blood cell fractions. Platelet and leukocyte counts were performed on all PRP samples using the same hematology analyzer (HemaTrue, Heska Corp, Loveland, CO, USA).

#### Sample Handling and Activation

Immediately after preparation, the PRP samples were divided; one portion of PRP was used for flow cytometry and the other portion was used for growth factor and TNF-α analysis.

#### Platelet Activation Status

For assessment of platelet activation, PRP was diluted to 5,000 platelets/μL using flow cytometry buffer solution (10 mM HEPES, 137 mM NaCl, 4mM KCl, 0.5 mM MgCl, 0.5 mM Na_2_HPO_4_, 0.1% glucose, 0.1% bovine serum albumin, pH = 7.4) immediately following preparation of the PRP. After dilution, the samples were divided into three activation treatment groups. CaCl_2_ (Haemonetics, Braintree, MA, USA) was added to the first aliquot to a final concentration of 20 mM, and HGT (Haematologic Corp., South Burlington, VT, USA) was added to the second aliquot to a concentration of 20 nM. Ten microliters of flow cytometry buffer solution were added to the third treatment group to serve as the unactivated control. Five microliters of Gly–Pro–Arg–Pro-NH_2_ (Sigma Corp, St. Louis, MO, USA) were added to each aliquot to prevent fibrin polymerization. Samples were gently mixed for 5 min at 23°C. After mixing, antibodies for CD61 (Clone Y2/51, Dako, Agilent Technologies, Santa Clara, CA; conjugated to allophycocyanin), CD62P (Clone AC1.2, Becton, Dickinson and Company, Franklin Lakes, NJ, USA; conjugated to phycoerythrin), or for platelet-bound fibrinogen using the Canine Activated Platelet-1 (CAP1)antibody (generously provided by M. Boudreaux) were added, followed by a 20-min incubation at 23°C in the dark. The CAP1 antibody was not conjugated to a fluorochrome, so a secondary antibody (IgG-FITC, Jackson Laboratories, West Grove, PA, USA) was added, followed by a second 20 min dark incubation. For both activated and unactivated samples, a separate aliquot was prepared with only the secondary antibody to reflect non-specific binding of fluorescent secondary antibody and to establish a defined threshold of positivity. Following these incubations, all samples were fixed by the addition of 2% formalin prior to analysis using a flow cytometer (Becton-Dickinson LSRII, Franklin Lakes, NJ, USA). The initial gating logic was derived based on the forward and side scatter plot of events that expressed CD61. Subsequent analysis counted 30,000 events in this platelet gate, and the median fluorescence intensity (MFI) was recorded from all samples for PE and FITC expression.

### Growth Factor and TNF-α Concentrations

Samples to be used for growth factor and TNF-α analysis were further divided into four groups that were activated with CaCl_2_ (final concentration of 20 mM), HGT (final concentration of 20 nM), or only had flow cytometry buffer solution added to serve as an unactivated control and to match the unactivated samples that were assessed using flow cytometry. The fourth aliquot was frozen at −80°C after PRP preparation and without additional centrifugation so that the platelets could be ruptured, or activated, by the freezing process. After addition of CaCl_2_, HGT, or buffer solution, samples were gently mixed on a platform rocker for 5 min at room temperature (22–24°C). Samples were subsequently centrifuged at 10,000 × *g* for 3 min to pellet any cellular contents and the supernatant was collected and frozen at −80°C until growth factor and cytokine concentrations were quantified (21). Aliquots from the four treatment groups were subsequently thawed at room temperature and TGF-β1, PDGF-BB, and TNF-α concentrations were quantified using ELISAs as specified above (30). All samples were run in duplicate, and all samples were assessed on a single 96-well plate in a single assay.

### Statistical Analysis

The distributions of TGF-β1 and PDGF-BB concentrations and the distributions of CD62P and CAP1 expression were not normally distributed based upon assessment of histograms, probability plots, and multiple tests for normality including a Shapiro–Wilk test (SAS version 9.4, Cary, NC, USA). Therefore, the effects of the different activation protocols on TGF-β1 and PDGF-BB concentrations and the expression of CD62P and CAP1 were assessed using a Friedman test (SAS version 9.4, Cary, NC, USA). Pair-wise comparisons were made using Conover’s test [R (R Core Team); http://www.R-project.org/] with a Bonferroni correction.

## Results

The platelet concentration of the different PRPs varied significantly (*p* < 0.0001) among the different preparation systems (Table 1). Tukey *post hoc* comparisons revealed that all systems were significantly different in direct system to system comparison (Table 1). The leukocyte concentrations of the different PRPs varied significantly (*p* < 0.0001) among the different PRP preparations (Table 1). Two systems produced a leukocyte-poor PRP with a leukocyte concentration just over 1 × 10^3^ cells/μL. Systems 3 and 4 notably increased leukocytes in comparison to the whole blood samples and system 5 had a neutral effect on leukocyte concentration in comparison to the whole blood (31). There were significant differences for all pair-wise comparisons among the different systems for leukocyte concentration except that there was no significant difference in the leukocyte concentrations between Systems 1 and 2. The percentage of leukocytes in the PRPs that were neutrophils for those systems with a notable leukocyte concentration were 42% for System 3, 39.4% for System 4, and 9.7% for System 5 (Table 1).

**Table 1:** **Mean (±SD) platelet and total leukocyte concentrations**.

Platelet-rich plasma-concentrating system					
	1	2	3	4	5
Platelets*	169,933 ± 74,460^a^	743,000 ± 301,719^c^	452,800 ± 185,747^b^	1,340,667 ± 285,520^e^	1,035,667 ± 514,614^d^
Leukocytes*	1,100 ± 600^a^	1,393 ± 1,128^a^	19,967 ± 4,936^c^	25,807 ± 6,993^d^	10,927 ± 4,894^b^
Neutrophils	0 (0–0.46)	0 (0–0.7)	7.3 (3.4–15.5)	8.0 (6.4–17.1)	0.8 (0–2.9)
Band neutr.	0 (0–0)	0 (0–0)	0 (0–0)	0 (0–0.8)	0 (0–0.2)
Monocytes	0 (0–0)	0 (0–0)	0 (0–0)	0 (0–1.5)	0 (0–0)
Lymphocytes	0.9 (0.4–1.8)	0.9 (0.3–3.5)	10.3 (5.0–14.9)	13.3 (5.6–23.9)	7.7 (2.9–13.9)
Eosinophils	0 (0–0.2)	0.05 (0–0.4)	0.8 (0–1.4)	2.2 (0–5.8)	0.6 (0–4.9)
Basophils	0 (0–0)	0 (0–0)	0 (0–0)	0 (0–0)	0 (0–0.2)

*Signifies statistical significant among the different systems (p < 0.0001).

*If two systems share a common superscript letter within a row, then they are not significantly different (*p* > 0.05). Platelet and total leukocyte concentrations are reported as cells/μL. The median (and range) are reported for the leukocyte subpopulations (neutrophils, band neutrophils, monocytes, lymphocytes, eosinophils, and basophils) in cells/μL × 10^3^*.

The anabolic growth factor concentrations of the different PRPs were based on PRPs from all 15 dogs for all systems except for System 2 in which 13 samples were assessed. Two samples had been frozen immediately following PRP preparation without an extra centrifugation to separate the platelets from the plasma in the PRP. Freezing can rupture platelets causing a release and associated increase in growth factor concentration and so these two samples were excluded (3). In addition, sufficient sample for quantifying TNF-α was available for just five PRPs from System 5. Since this system produced the smallest volume of PRP there was insufficient sample with which to quantify all growth factors and TNF-α concentrations for all 15 PRP samples.

The TGF-β1 and PDGF-BB concentrations of the PRPs prepared from different systems differed significantly (*p* < 0.0001; Table 2). Transforming growth factor-β1 (lower limit of quantification 31.3 pg/mL) was present in measurable concentrations in all samples and was significantly higher in samples that had been activated by CaCl_2_ (System 2) in comparison to the PRP from all other systems based upon pair-wise comparisons (*p* < 0.0001). There were also significant differences (*p* < 0.001) among the other systems in which platelet activation had not been performed and are depicted in Table 2.

**Table 2:** **Median (range) growth factor and cytokine concentrations (pg/mL)**.

PRP-concentrating system					
	1	2	3	4	5
TGF-β1	2,633 (1,331–8,444)^a^	27,913 (16,450–47,782)^d^	4,830 (90–15,671)^b,c^	4,042 (2,242–6,404)^a,b^	6,296 (3,878–17,703)^c^
PDGF-BB	0 (0–0)^a^	3,072 (737–6,562)^b^	47 (0–270)^a^	0 (0–296)^a^	0 (0–57)^a^
TNF-α	0 (0–40.8)	0 (0–0)	0 (0–0)	0 (0–0)	0 (0–0)
VEGF	0 (0–0)	0 (0–0)	0 (0–0)	0 (0–0)	0 (0–0)

*Statistical comparisons were made between the different platelet-rich plasma (PRP) systems for transforming growth factor-β1 (TGF-β1) and platelet-derived growth factor-BB concentrations. If two systems share a common superscript letter within a row they are not significantly different (*p* > 0.05)*.

Platelet-derived growth factor-BB was detectable only in samples that had been activated by CaCl_2_ (System 2; lower limit of quantification 31.3 pg/mL). Accordingly, the concentration of PDGF-BB was statistically greater in the releasate from this system when compared to all other systems (*p* < 0.0001). The concentration of VEGF was below the lower limit of quantification (39.1 pg/mL) for the assay in all samples. Similarly, the concentration of TNF-α was below the lower limit of quantification (7.8 pg/mL) for all samples except for three of the samples from System 1. As a result, no correlations between any cellular populations in the PRPs and VEGF or TNF-α were assessed.

**Figure 1:**
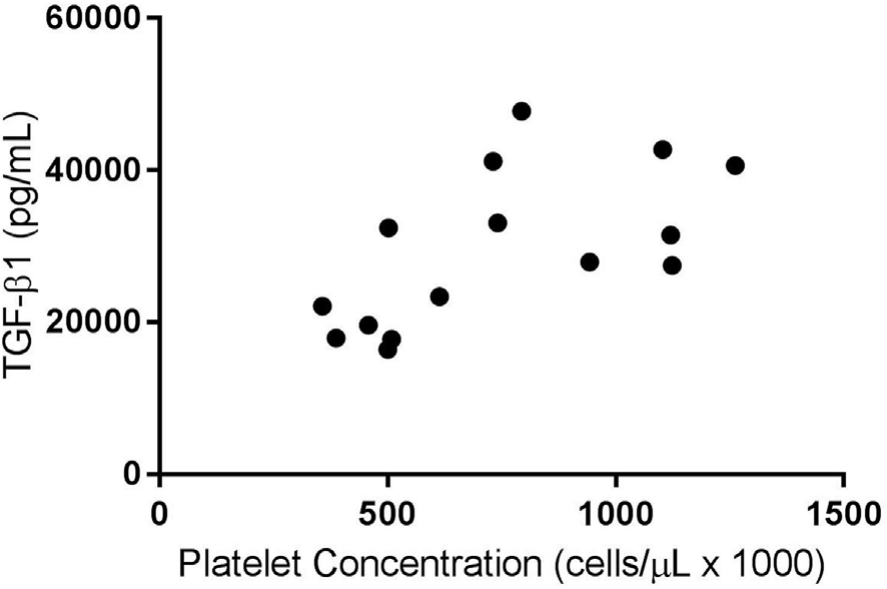
**Correlation of platelet and transforming growth factor-β1 (TGF-β1) concentrations for activated platelet-rich plasmas (PRPs) from System 2 (*R*^2^ = 0.40; *p* < 0.05)**.

**Figure 2:**
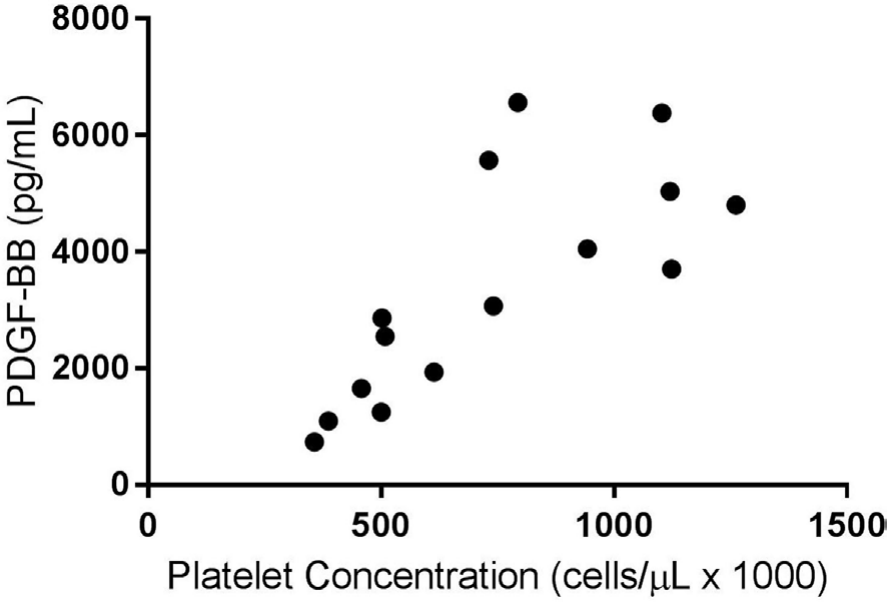
**Correlation of platelet and platelet-derived growth factor-BB (PDGF-BB) concentrations for activated platelet-rich plasma (PRPs) from System 2 (*R*^2^ = 0.58; *p* < 0.05)**.

A weak positive correlation was found between the platelet concentration of PRPs that were not activated and circulating TGF-β1 concentration (*R*^2^ = 0.07, *p* < 0.05). When only the PRP releasate from system 2 was assessed, there was a moderate positive correlation between the platelet concentration and the concentration of TGF-β1 (*R*^2^ = 0.40, *p* < 0.05, Figure 1) and a stronger correlation between platelet concentration and PDGF-BB concentrations (*R*^2^ = 0.58, *p* < 0.05, Figure 2).

### Effects of Platelet Activation

The mean (±SD) platelet concentration in the PRP (Angel, Arthrex Vet Systems, Naples, FL, USA) samples was 969 × 10^3^/μL (±355 × 10^3^/μL), with a 4.2 (±1.3)-fold mean increase in platelet concentration above the baseline whole blood sample, and with a mean leukocyte concentration of 7.24 × 10^3^ leukocytes/μL (±0.004 × 10^3^/μL). The prothrombin time, activated partial thromboplastin time, and fibrinogen values were within reference ranges for the nine dogs for which these values were quantified.

**Figure 3:**
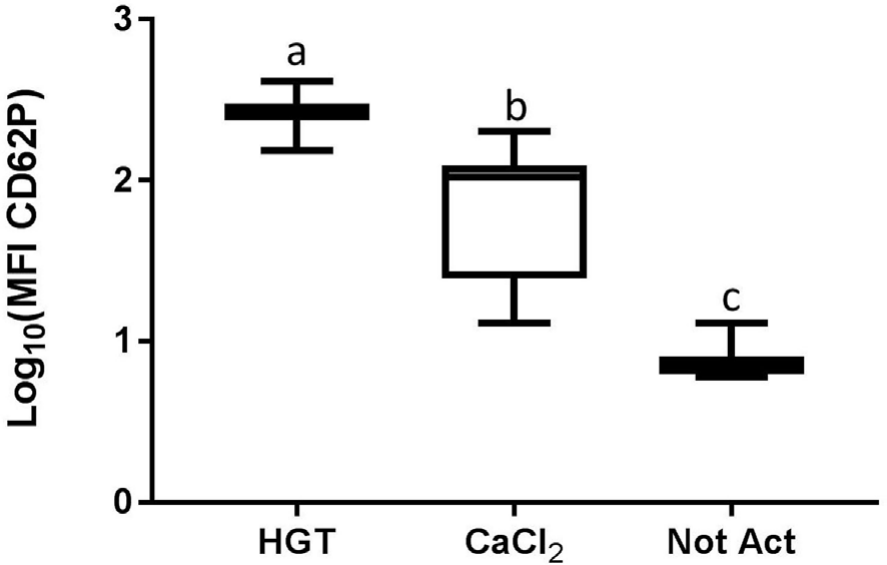
**Median fluorescence intensity (MFI) for CD62P**. HGT, human γ-thrombin; CaCl_2_, calcium chloride; Not Act, not activated. The data have been log transformed to reduce skew of the data for visualization of differences among the three groups. The middle line represents the median, the ends of the box are the 25th and 75th percentiles, and the whiskers are the minimum and maximum values. Activation treatment group had a significant effect on MFI (*p* < 0.0001), and each of the treatment groups was significantly different than the other treatment groups based upon pair-wise comparison.

**Figure 4:**
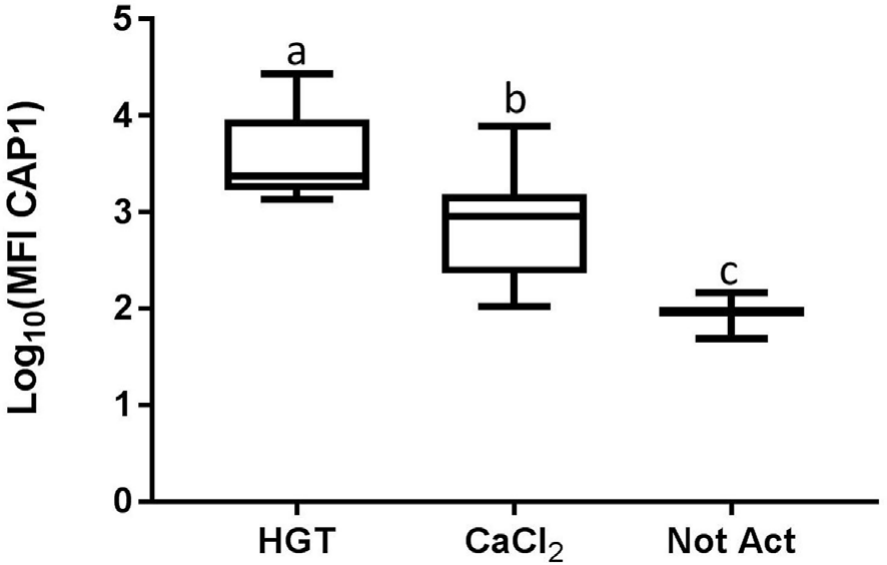
**Median fluorescence intensity (MFI) for CAP1**. HGT, human γ-thrombin; CaCl_2_, calcium chloride; Not Act, not activated. The data have been log transformed to reduce skew of the data for visualization of differences among the three groups. The middle line represents the median, the ends of the box are the 25th and 75th percentiles, and the whiskers are the minimum and maximum values. Activation treatment group had a significant effect on MFI (*p* < 0.0001) using a repeated measures analysis and each of the treatment groups was significantly different than the other treatment groups based upon pair-wise comparison.

**Figure 5:**
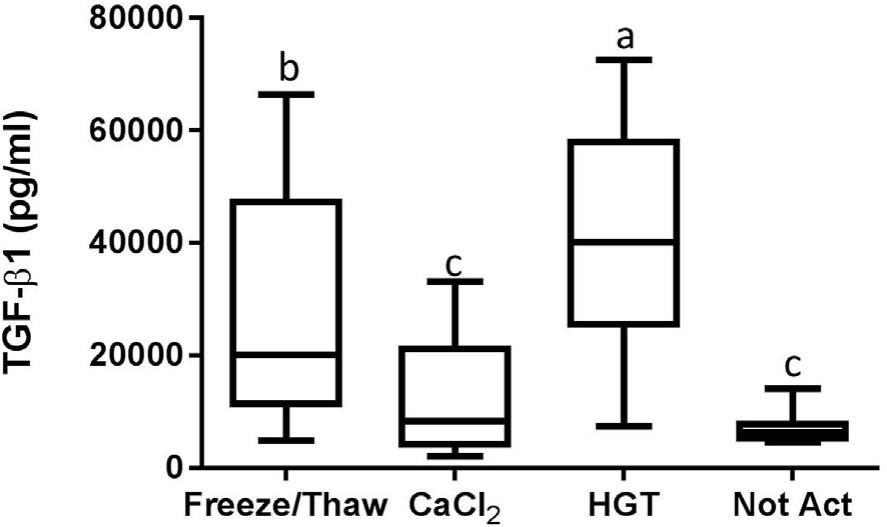
**Transforming growth factor-β1 (TGF-β1) concentration**. HGT, human γ-thrombin; CaCl_2_, calcium chloride; Not Act, not activated. The middle line represents the median, the ends of the box are the 25th and 75th percentiles, and the whiskers are the minimum and maximum values. Activation treatment group had a significant effect on TGF-β1 concentration (*p* < 0.01). If two groups share a common letter they are not significantly different (*p* > 0.05).

**Figure 6:**
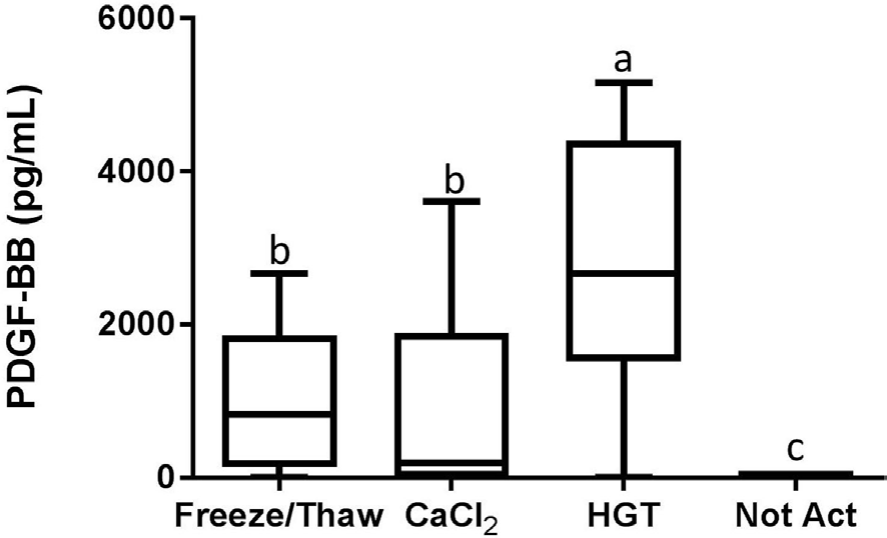
**Platelet-derived growth factor-BB (PDGF-BB) concentration**. HGT, human γ-thrombin; CaCl_2_, calcium chloride; Not Act, not activated. The middle line represents the median, the ends of the box are the 25th and 75th percentiles, and the whiskers are the minimum and maximum values. Activation treatment group had a significant effect on PDGF-BB concentration (*p* < 0.001). If two groups share a common letter they are not significantly different (*p* > 0.05).

Based upon a Friedman test the activation treatment had a significant effect on CD62P MFI (*p* < 0.0001; Figure 3), CAP1 MFI (*p* < 0.0001; Figure 4), TGF-β1 concentration (*p* < 0.01; Figure 5), and PDGF-BB concentration (*p* < 0.001; Figure 6). For each of these outcome variables, the degree of platelet activation, based upon either MFI or growth factor concentration, was numerically greatest with HGT activation, intermediate with CaCl_2_ activation or freeze/thaw, and lowest with the unactivated samples (Figures 3–6). With pair-wise comparisons using a Conover’s test and Bonferroni correction, HGT activation resulted in significantly higher CD62P expression than CaCl_2_ activation (*p* < 0.0001), and CaCl_2_ activation resulted in significantly greater CD62P expression than the unactivated samples (*p* < 0.0001, Figure 3). Treatment had a similar and significant effect on CAP1 MFI (*p* < 0.0001; Figure 4), and based upon pair-wise comparisons MFI with HGT was significantly greater than with CaCl_2_ activation (*p* < 0.0001) and MFI with CaCl_2_ activation was significantly greater than the unactivated control (*p* < 0.0001; Figure 4).

Detectable concentrations of TGF-β1 and PDGF-BB were identified in multiple samples, while TNF-α was not detected in any of the samples. There were statistically significant differences among the activation groups for both TGF-β1 and PDGF-BB concentrations (*p* < 0.01 and *p* < 0.001, respectively; Figures 5 and 6). The TGF-β1 concentration was significantly greater with HGT activation than with all other treatment groups including with a freeze/thaw (*p* < 0.05). TGF-β1 concentration with freeze/thaw was greater than that with CaCl_2_ activation (*p* < 0.05) or the unactivated sample (*p* < 0.001). The TGF-β1 concentration was not statistically significantly different between the CaCl_2_ and unactivated control (*p* > 0.05; Figure 5). The PDGF-BB results were similar to the TGF-β1 results. The PDGF-BB concentration was greatest with HGT activation, intermediate with freeze/thaw and CaCl_2_ activation and not significantly different between these two groups (*p* > 0.05), and significantly lower than either freeze/thaw (*p* < 0.0001) or CaCl_2_ activation (*p* < 0.001; Figure 6) with the unactivated control.

## Discussion

These data demonstrate substantial variability among the platelet, leukocyte, and growth factor concentrations of different canine PRPs. Variability in the leukocyte concentrations of different canine, equine, and human PRPs has been shown previously so these results were not unexpected (1, 4, 12, 16). In addition, the data from this study demonstrate that the specific leukocyte populations in the different PRPs also differed dramatically depending upon the method of PRP preparation. Specifically, PRP made with systems 3, 4, and 5, all had a notable leukocyte concentration. However, approximately 40% of the leukocytes in the PRPs made with Systems 3 and 4 were neutrophils, while approximately 10% of the leukocytes in the PRP from System 5 were neutrophils and approximately 80% of the leukocytes in that PRP were lymphocytes. Similar results were identified with another study of canine PRPs in which neutrophils comprised a substantial proportion of the leukocytes in PRPs made using System 3 and 4 (16). In addition, that study found that one of the PRP preparation systems produced a PRP that had a substantial leukocyte population, few neutrophils, and consisted primarily of monocytes and lymphocytes (16). The similarity in data from this study and the previously published study regarding PRPs made from Systems 3 and 4 provides re-assuring evidence that these results specifically are repeatable (16). Furthermore, that both this study and previous study document production of a PRP in which the leukocyte population is largely devoid of neutrophils and comprised primarily of lymphocytes and monocytes is also re-assuring that such PRPs can be produced with canine blood (16). The mechanism by which System 5 from this study produced a PRP with a leukocyte content that consisted primarily of mononuclear cells, whereas Systems 3 and 4 produced PRPs containing a greater concentration of neutrophils, remains unclear. However, this finding is potentially pertinent as studies of human PRPs demonstrated significant positive correlations between neutrophil and macrophage concentrations and the inflammatory cytokines IL-1β and matrix metalloproteinase-9 (4). Further, neutrophils have been hypothesized to impede wound healing (20). Conversely, the authors are not aware of any established association between lymphocytes and catabolic cytokine concentrations in PRPs.

As has been found with human PRPs, these data also demonstrate positive correlations between platelet concentration in PRP and anabolic growth factor concentrations. However, the correlation between platelet concentration and TGF-β1 was weak, and was non-existent for PDGF-BB, when assessing the PRPs that had not been activated. Conversely, the correlations between platelet concentration and TGF-β1 and PDGF-BB concentrations were moderate or strong for activated PRP samples. This difference is most likely explained by the intentional activation of these PRP samples. Although this could be perceived as a flaw in study design, and thus an unfair comparison among systems, all the samples in this study were handled as advised by the manufacturers. Hence, the results are representative of the PRPs obtained in clinical practice when following manufacturer instructions. More importantly, these data suggest that platelet activation was more influential on anabolic growth factor concentration than the platelet concentration alone. In turn, it is relevant to assess whether the platelets in all PRPs are capable of activation and growth factor release and how platelets in different canine PRPs respond to different activators (endogenous or exogenous).

To this end, PRP samples were then made with one of these commercial systems using a new group of canine blood donors. These PRP samples were assessed for the effects of activation protocol on platelet activation and growth factor release. Importantly, the PRP preparation methodology did not result in platelet activation, indicated by low baseline CD62P expression or platelet-bound fibrinogen in unactivated samples. Furthermore, growth factors and TNF-α concentrations were minimal or absent in the PRP samples that were not activated. Conversely, HGT provided a robust platelet activation as CD62P, platelet-bound fibrinogen, and TGF-β1 and PDGF-BB concentrations were greatest with HGT activation (Figures 3–6). These results are at least similar to one study with dog PRP demonstrating substantial CD62P upregulation with bovine thrombin activation (25).

Use of CaCl_2_ also resulted in platelet activation, albeit to lesser degree than HGT activation. There was greater platelet expression of CD62P and more platelet-binding of fibrinogen (CAP1) when samples were activated with CaCl_2_ when compared to unactivated samples (Figure 3). In addition, PDGF-BB concentrations in the PRP samples activated with CaCl_2_ were significantly greater than those in the unactivated samples. There was no significant difference between the CaCl_2_-activated samples and unactivated samples with regarding to TGF-β1 concentration in this study. These results are somewhat similar to a previous study that quantified TGF-β1 in canine PRP and found that activation with calcium gluconate increased TGF-β1 concentrations in comparison to unactivated controls (19). The studies are similar in that they both demonstrate positive effects of calcium activation on canine PRP, yet they differ with respect to a significant effect on TGF-β1 concentration specifically (19). We hypothesize that the difference in effect on TGF-β1 release is attributable to a Type II statistical error in this study, in part because of use of fewer dogs, non-normality of the TGF-β1 concentrations, and use of a non-parametric statistical test. The previously performed study used more dogs (16 rather than 12) and they concluded their data were normally distributed and performed a parametric test, thus likely providing greater statistical power (19).

A single freeze/thaw cycle similarly caused an increase in the concentration of both TGF-β1 and PDGF-BB when compared to aliquots that were not activated. The concentration of TGF-β1 was also greater with a single freeze/thaw than with CaCl_2_ activation. Freezing the PRP may have increased growth factor concentration by cold-induced platelet activation occurring during the freezing process (32). Alternatively, the increase in growth factor concentration was probably caused at least in part by rupture of the platelets and granules during freezing and thawing, allowing release of growth factors (3).

When considered together, these data demonstrate that platelet activation status has a profound impact on the release of anabolic growth factors from canine PRPs, more so than just the cellular composition of the PRP. A relevant question is whether activation of PRP should be performed when used clinically. Exogenous activation may not be necessary as exposure to collagen, such as may occur in injured soft tissue structures, can also result in platelet activation (22, 23). Furthermore, although initial growth factor release with equine and human PRPs tends to be less with collagen than thrombin or calcium *in vitro*, at least one study demonstrated greater cumulative *in vitro* TGF-β1 release with collagen exposure than thrombin (22–24). Therefore, injection of PRP that has not been exogenously activated may result in sustained release of growth factors from platelets as they are activated at the site of injury (23).

Although use of PRP that has not been exogenously activated may result in sustained activation *in vivo*, this is difficult to confirm and *in vivo* activation may not occur consistently (21). To ensure provision of the highest concentration of growth factors at the time of PRP administration, these data support the conclusion that thrombin (HGT in this study) should be used to activate the canine PRPs evaluated in this study. However, the availability, cost, and potential reaction to a xenogenic protein should also be considered. Conversely, use of calcium (either chloride or gluconate) provides a more moderate activation, but does not involve administration of a xenogenic protein. Last, if one were to freeze the PRP (Angel, Arthrex Vet Systems, Naples, FL, USA), injection of the thawed solution would result in delivery of a solution containing TGF-β1 and PDGF-BB concentrations that are as great or greater than that obtained with CaCl_2_ activation (Figures 5 and 6). However, numerous studies demonstrate that freezing is detrimental to platelet morphology, function, and growth factor release (33–35). As a result, platelets in previously frozen PRP are unlikely to continue synthesizing growth factors following thawing (33–35). Conversely, continued synthesis of platelet-derived proteins following activation, rather than just release of what growth factors are within the α-granules at the time of activation, has been shown *in vitro* (21, 36). Therefore, application of fresh PRP may allow continued production and release of growth factors *in vivo* for an extended period of time.

In summary, there are notable differences among the PRPs produced using these five different commercially available systems. Such differences include substantial variability in platelet, neutrophil, monocyte, and lymphocyte concentrations as well as significant differences in anabolic growth factor concentrations. As with human PRPs, there are positive correlations between platelet concentrations and TGF-β1 and PDGF-BB concentrations in canine PRPs. However, comparison of growth factor concentrations among the different canine PRPs suggest that intentional exogenous activation has a greater effect than cellular composition on growth factor content and release in the PRP. This hypothesis is supported by the data comparing the effects of different activation protocols on CD62P expression, platelet binding of fibrinogen, and growth factor release using one canine PRP (Angel, Arthrex Vet Systems, Naples, FL, USA). The baseline platelet activation and growth factor concentrations were low with this PRP (in both parts of this study) when activation was not performed. However, activation with HGT caused a significant increase in CD62P expression, platelet binding of fibrinogen, and release of TGF-β1 and PDGF-BB. A more moderate response was noted with use of CaCl_2_. A freeze/thaw of this PRP increased the concentration of both TGF-β1 and PDGF-BB in comparison to samples that were not activated and provided a higher concentration of TGF-β1 than the CaCl_2_ activated samples. Ideally the assessment of intentional platelet activation would be performed with all canine PRP preparations to confirm that platelets in these different preparations are capable of activation and growth factor release. However, the data from this study and others suggest that most platelets in canine PRPs do respond to intentional activation (19, 25). A remaining clinical question is whether canine PRPs should be activated prior to use or whether activation occurs consistently *in vivo*.

## Author Contributions

SF contributed to experimental design, funding acquisition, data collection, and prepared the manuscript. KB performed data collection and assisted with manuscript preparation. AS assisted with data collection. BG and BB assisted with experimental design, data collection, and manuscript editing.

## Conflict of Interest Statement

SF is a consultant for Arthrex Vet Systems. The other authors declare no conflict of interest.
